# Low-frequency blood flow oscillations in congestive heart failure and after β1-blockade treatment

**DOI:** 10.1016/j.mvr.2008.07.006

**Published:** 2008-11

**Authors:** A. Bernjak, P.B.M. Clarkson, P.V.E. McClintock, A. Stefanovska

**Affiliations:** a Faculty of Electrical Engineering, University of Ljubljana, Slovenia; b Cardiology Department, Royal Lancaster Infirmary, LA1 4RP, UK; c Physics Department, Lancaster University, LA1 4YB, UK

**Keywords:** Blood flow oscillations, Congestive heart failure, β_1_-blockers, Iontophoresis, Laser Doppler flowmetry, Wavelet transform, Logarithmic frequency resolution, Dynamics

## Abstract

Laser Doppler flowmetry (LDF) of forearm skin blood flow, combined with iontophoretically-administered acetylcholine and sodium nitroprusside and wavelet spectral analysis, was used for noninvasive evaluation of endothelial function in 17 patients newly diagnosed with New York Heart Association class II–III congestive heart failure (CHF). After 20 ± 10 weeks' treatment with a β_1_-blocker (Bisoprolol), the measurements were repeated. Measurements were also made on an age- and sex-matched group of healthy controls (HC). In each case data were recorded for 30 min. In HC, the difference in absolute spectral amplitude of LDF oscillations between the two vasodilators manifests in the frequency interval 0.005–0.0095 Hz (*p* < 0.01); this difference is initially absent in patients with CHF, but appears following the β_1_-blocker treatment (*p* < 0.01). For HC, the difference between the two vasodilators also manifests in normalised spectral amplitude in 0.0095–0.021 Hz (*p* < 0.05). This latter difference is absent in CHF patients and is unchanged by treatment with β_1_-blockers. It is concluded that there are two oscillatory skin blood flow components associated with endothelial function. Both are reduced in CHF. Activity in the lower frequency interval is restored by β_1_-blocker treatment, confirming the association between CHF and endothelial dysfunction but suggesting the involvement of two distinct mechanisms.

## Introduction

Congestive heart failure (CHF) is a complex clinical condition, in which the history of its management reflects the evolving understanding of its pathophysiology. CHF is associated with a typical neurohormonal response involving activation of both the renin–angiotensin system and the sympathetic nervous system. This activation is deleterious and current therapeutic strategies to block the effects of this activation e.g. with angiotensin converting enzyme inhibitors (Eichhorn and Bristow, 1996) and β-adrenergic blocking agents (Bristow, 2000; López-Sendón et al., 2004) have been clearly demonstrated to benefit favourably the poor outcome in all grades of severity of the condition. The results seen with β-blockade are particularly effective in producing a long-term reduction in mortality from both sudden death, and progressive cardiac failure, and improving symptomatology. Despite 30 years of research the mode of action of β-blockers is still incompletely understood. It is likely that they act through multiple mechanisms including reducing tendencies to malignant dysrhythmias, and favourable effects on reverse remodeling (Silke, 2006; Udelson, 2004).

The endothelium plays a pivotal role in regulating blood flow by releasing relaxing and constricting factors, a role that has been shown to be impaired in CHF (Drexler et al., 1992; Vanhoutte, 1996; Morgan et al., 2004). One of the mechanisms by which this occurs is through decreased peripheral production of endothelium-derived nitric oxide (NO) (Katz et al., 1999; Zhao et al., 1996), possibly because of reduced shear stress due to reduced peripheral perfusion (Drexler, 1998). Endothelial dysfunction in CHF has recently been shown to be associated with an increased mortality risk (Katz et al., 2005). In isolated cellular models, direct effect of β-blockers has been shown on endothelial function and was suggested to at least partially explain the efficacy of beta-blockers in the treatment of advanced heart failure (Garlichs et al., 1999).

Various techniques are available to evaluate endothelial function, including brachial arterial imaging (Kuvin and Karas, 2003), or plethysmography and laser Doppler flowmetry (LDF) (Cracowski et al., 2006) that monitor vasodilatory responses to the administration of an endothelial-dependent vasodilator such as acetylcholine (ACh). A decreased response to ACh but not to the endothelial-independent vasodilator sodium nitroprusside (SNP) is considered evidence of endothelial dysfunction. Besides basal differences in responses to these two substances, LDF enables study of the oscillatory components in the blood flow (Kvernmo et al., 1999; Stefanovska et al., 1999; Kvandal et al., 2003; Stefanovska and Bračič, 1999). Using LDF it was intended to examine, in particular, those blood flow frequencies that are known to reflect endothelial reactivity.

The study was motivated by the perception of the cardiovascular system in terms of at least 5 distinct coupled oscillatory processes with different frequencies. The state of the system can be characterised by the oscillatory amplitudes, and by the couplings between the oscillators. Heart rate variability arises because lower frequency oscillations (especially, but not only, respiration) are coupled to the cardiac rhythm. Unlike many earlier studies, our interest therefore centres on blood flow *dynamics* (Stefanovska and Bračič, 1999), rather than on basal values. The main advantage of this approach is that its frequency resolution enables the contributions from different physiological processes to be distinguished, and it has already been applied to other disease states related to the cardiovascular system: type 2 diabetes (Urbančič-Rovan et al., 2004) and post acute myocardial infarction (Ažman-Juvan et al., 2008). It was postulated that the difference in endothelial reactivity of the CHF group and an age-matched healthy control (HC) group will be manifested in particular oscillatory components. The mechanism of action of β_1_-blockers in CHF is not fully understood, so the final aim of the study was to evaluate the effects that β_1_-blockers have on blood flow dynamics in CHF.

## Methods

### Subjects

Patients for the CHF group were recruited either from cardiology clinics in the Royal Lancaster Infirmary or from patients referred directly for open access echocardiography. None of them exhibited significant edema. The severity of heart failure was established via echocardiography. Left ventricular diameter and left ventricular ejection fraction (LVEF) were determined. Inclusion criteria were that they had LVEF < 35% and symptoms between class II–III of the New York Heart Association (NYHA) classification. Exclusion criteria were recent myocardial infarction, or cerebrovascular accident (within 6 months), fibrillation, other life threatening co-morbidity, advanced frailty, current use of β-blocking drugs, or contra-indications to β-blocker therapy. All but one CHF patients remained on established treatment with angiotensin converting enzyme inhibitors and diuretics throughout the study; the exception was on an angiotensin-receptor antagonist and diuretics.

After the initial set of measurements all CHF patients were then treated with Bisoprolol (a selective β_1_-antagonist). This was commenced in a dose of 1.25 mg under direct observation, and then increased, at intervals of a minimum of 1 week, through 2.5 mg, 3.75 mg and 5 mg. Finally it was increased at intervals of a minimum of 4 weeks from 7.5 mg to 10 mg. Upwards titration was stopped if symptomatic hypotension, or pulse rate < 50/min, or side-effects of the β-blocker, appeared. A second set of measurements was taken after 5 weeks of a stable dose (β-CHF subject group) and 20 ± 10 weeks after the first set of measurements. LVEF and NYHA were determined only before treatment. Patients' data are summarized in Table 1.

Healthy age (66 ± 6 years) and sex (8 F and 13 M) matched control subjects were recruited from the local community. None of them was on medication or had a history of cardiovascular disease or problems related to the cardiovascular system, including hypertension or hypercholesterolemia. A single set of measurements, identical to that recorded for CHF patients, was taken from each of them.

All participants gave their informed consent in writing. The investigation conformed with the principles outlined in the Declaration of Helsinki and was approved by the Local Ethics Committee of the Morecambe Bay Hospitals Trust.

### Measurements

Subjects lay supine on a bed and relaxed for 15 min prior to the commencement of recording. Peripheral blood flow, heart rate, respiration, and skin temperature were simultaneously recorded for 30 min, based respectively on laser Doppler flowmetry (LDF), a conventional 3-lead ECG, a Biopac respiratory effort transducer placed around the thorax, and Thermilinear temperature sensors (YSI Inc, Ohio, USA) placed on the arm and leg. The ECG, respiration and temperature signals were amplified using a specially designed signal conditioning unit (Cardiosignals, Jožef Stefan Institute, Slovenia). Signals were digitized at 400 Hz with 16-bit resolution by use of a National Instruments PCI-6035E A/D converter, and stored in a personal computer. The temperature of the room was maintained at 21 ± 2 °C.

### Laser Doppler flowmetry

Skin blood flow signals were measured by the laser Doppler perfusion technique using a DRT4 LDF monitor (Moor Instruments Ltd, Axminster, UK). Two MPI–V2 probes were mounted within MIC1–IONlr chambers, where the vasoactive substances were inserted for iontophoretic administration in the same area as that where the blood flow was being recorded. Measurements were made on the volar aspect of the right forearm, choosing areas of skin that were free from blemishes. Both probes were positioned in areas with similar vasculature and without larger vessels in their vicinity. For each set of measurements the two chambers were placed 2 cm apart with random relative orientation. Near-infra-red light (wavelength 780 nm) was delivered to the probes via a nearly loss-free optical fibre. The back-scattered light was collected and returned to the monitor via another optical fibre, where it was converted to an analogue electrical signal. The cut-off frequency of the low-pass filter was 22.5 kHz, and an output time constant of 0.1 s was selected. The LDF probes were calibrated against a flux standard (Moor Instruments) and the blood flow was expressed in arbitrary units (AU).

### Iontophoresis

**Fig. 1:**
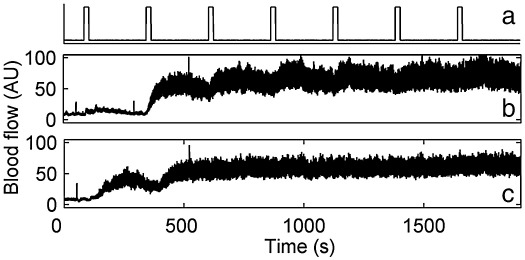
Simultaneously measured LDF signals showing how blood flow changes in response to iontophoresis with the two vasodilators. (a) Timing of the 100 μA iontophoresis current pulses; (b) blood flow in response to ACh; and (c) in response to SNP.

Two vasodilators were iontophoretically applied: ACh whose action is endothelium-dependent, and SNP an endothelium-independent direct NO donor. The difference in the blood flow enhancements that they produce in any oscillatory component is related to the responsiveness of the vascular endothelium (Kvernmo et al., 1999; Stefanovska et al., 1999; Kvandal et al., 2003; Stefanovska and Bračič, 1999). The 1% ACh and SNP solutions were prepared on the day of the study in each case. They were drawn through the cutaneous barrier at each of the LDF measuring sites by means of a constant electrical current of 100 μA. A Moor Instruments MIC1-e current controller was used. The iontophoresis current was applied in 7 pulses of 20 s, with a separation of 240 s between each, as indicated in Fig. 1a. Fig. 1b,c shows examples of blood flow responses to ACh and SNP for a healthy subject. They illustrate the large increase of oscillatory activity that typically occurs in addition to the increased average flow.

### Data analysis

The time averages of the signals from the LDF probes were calculated, and the signals were then resampled at 10 Hz using a moving average technique. Trends were removed by giving the moving average a window length of 200 s, thereby eliminating frequencies below 0.005 Hz. Following this pre-processing, the wavelet transform (using the Morlet mother wavelet (Morlet, 1983)) was computed to analyse the frequency content of the signals. The advantages in comparison to conventional Fourier techniques include the logarithmic frequency resolution, which enables an extremely wide range of frequencies to be accommodated: in the present case, the characteristic frequencies of oscillations in the LDF signals differed by a factor > 100. All data analyses including the wavelet transform and data presentation were performed using code written within Matlab (The MathWorks, Inc.).

**Fig. 2:**
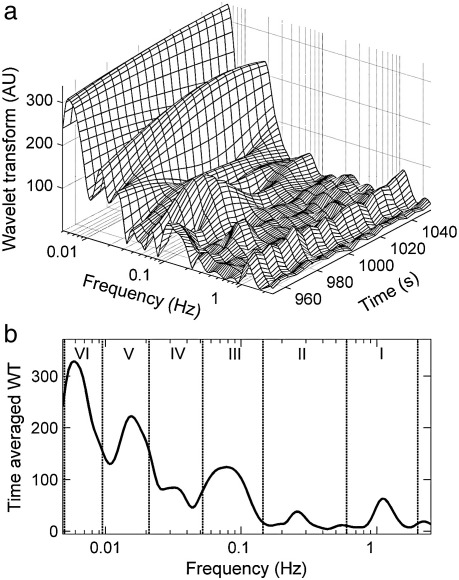
(a) The wavelet transform of an LDF skin blood flow signal, illustrating the presence of distinct spectral peaks whose frequencies and amplitudes vary in time. The wavelet coefficients, presented in the time–frequency domain, were calculated from the basal flow of a healthy subject at rest. Only a short time section of the transform is presented. (b) A time-average of the wavelet transform showing the division of the frequency scale into six intervals.

Computation of the wavelet transform of the blood flow signal yields the usual 3-dimensional structure above the time–frequency plane, exhibiting clearly resolved spectral peaks whose positions vary in time (Stefanovska et al., 1999; Stefanovska and Bračič, 1999) as shown in Fig. 2a, and a time-average as shown in Fig. 2b. The positions of the spectral peaks also differ slightly from subject to subject. In earlier work, based on 20-minute recordings, five frequency intervals were defined (Stefanovska et al., 1999; Stefanovska and Bračič, 1999) such that each of them contains only one peak: 0.0095–0.021, 0.021–0.052, 0.052–0.145, 0.145–0.6, and 0.6–1.6 Hz. They are attributed respectively to NO-dependent endothelial (Kvernmo et al., 1999; Stefanovska et al., 1999; Kvandal et al., 2003, 2006; Stewart et al., 2007), neurogenic (Kastrup et al., 1989; Söderström et al., 2003; Landsverk et al., 2006), myogenic (Kvernmo et al., 1999; Johnson, 1991; Bertuglia et al., 1994), respiratory and cardiac processes, as summarized in Table 2. The lowest detectable frequency component depends on the length of the signal and therefore longer recordings enabled investigations of blood flow spectrum below 0.0095 Hz. In the study of Kvandal et al. 30-minute LDF recordings were performed and an additional spectral peak centred near 0.007 Hz was observed and defined as the sixth frequency subinterval 0.005–0.0095 Hz (Kvandal et al., 2006). The physiological origin of this oscillation was investigated in the latter study and was shown to be related to endothelial activity, but not related to NO or prostaglandins. Therefore an endothelial regulatory process, different from that in the 0.0095–0.021 frequency interval, has been suggested.

These six spectral peaks have recently been confirmed in an independent study (Hafner et al., 2007) based on wavelet transformation of LDF data from healthy subjects and are also investigated in the present study.

For convenience, the frequency intervals are labeled I–VI as specified in Table 2 and indicated in Fig. 2b. Note that, compared to earlier work (Stefanovska and Bračič, 1999), the intervals are redesignated, now numbering them starting from the high frequency end, in order to reflect the probable future discovery of oscillatory components at even lower frequencies. In addition, to allow for the increased heart rate in CHF patients, the 1.6 Hz boundary value of the heartbeat-related frequency range (interval I) has been increased to 2 Hz. Detailed description of all the oscillations and their physiological origin is presented in the Appendix A.

Two measures are used to quantify the contribution of the oscillations to the total blood flow: their absolute and normalised spectral amplitude. The word ‘spectral’ indicates they are both calculated from the coefficients of the wavelet transform. Absolute spectral amplitude is the mean value of the wavelet transform, and was calculated separately within each frequency interval. Absolute spectral amplitude over the whole frequency range from 0.005 to 2 Hz was also calculated. To differentiate it from absolute amplitudes within specific intervals, it was introduced as average spectral amplitude. The normalised spectral amplitudes are calculated from the absolute spectral amplitudes and are defined as the ratio between the absolute spectral amplitude within a given subinterval and the average spectral amplitude.

R-peaks in ECG signals were automatically detected and manually checked, and the corresponding instantaneous heart rate (IHR) time series was generated, composed of inverse values of R–R periods. The mean heart rate (HR) for each subject was then obtained as a time-average of the IHR signal and the heart rate variability was defined as its standard deviation. In addition, maximal values in the respiration signals were automatically detected, manually checked, and analysed in a similar manner to yield the instantaneous respiration frequency (IRF) time series. The mean respiratory frequency (RF) was obtained as a time-average of IRF and the respiratory frequency (RF) variability was introduced as the standard deviation of IRF.

The analysis was blinded, in that the relevant researcher had no connection with the experiments, but simply analyzed the anonymized time-series data with which he was provided.

### Statistical analysis and presentation

**Fig. 3:**
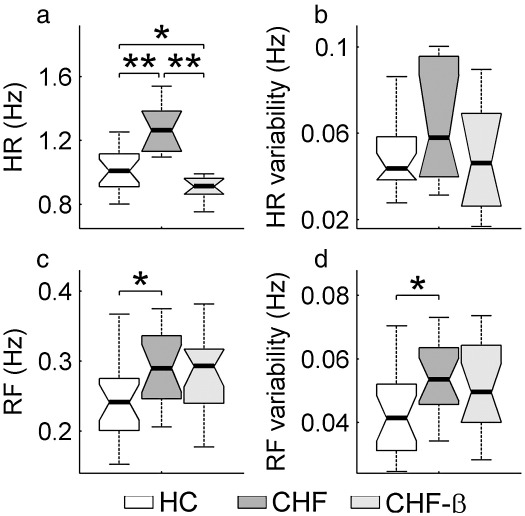
Summary of heart and respiration frequencies (a and c) and their variability (b and d) for the three subject groups. 0.01 < *p* < 0.05 is indicated by ⁎ and *p* < 0.01 by ⁎⁎. For an explanation of data presentation and error bars, see text (subsection on statistical analysis and presentation).

**Fig. 4:**
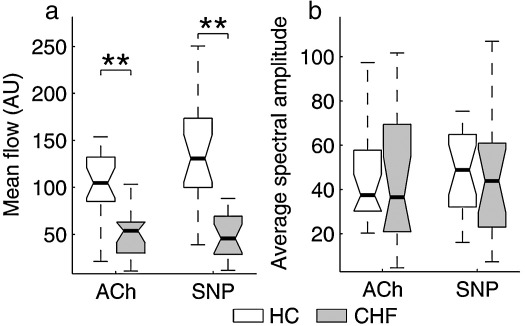
The effects of ACh and SNP on the mean value of the blood flow signal and the average spectral amplitude for the CHF and HC subject groups. *p* < 0.01 is indicated by ⁎⁎. For an explanation of data presentation and error bars, see text (subsection on statistical analysis and presentation).

**Fig. 5:**
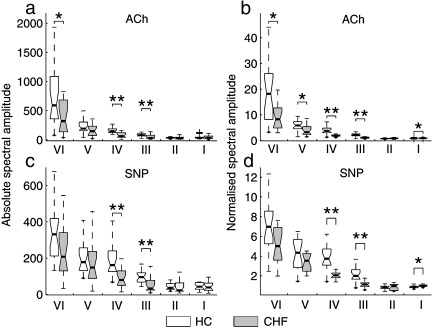
The effects of ACh and SNP on the individual oscillatory components in blood flow for the CHF and HC subject groups. (a) Effect of ACh on the absolute spectral amplitude; (b) its effect on the normalised spectral amplitude; (c) effect of SNP on the absolute spectral amplitude; (d) its effect on normalised spectral amplitude. 0.01 < *p* < 0.05 is indicated by ⁎ and *p* < 0.01 by ⁎⁎. For an explanation of data presentation and error bars, see text (subsection on statistical analysis and presentation).

**Fig. 6:**
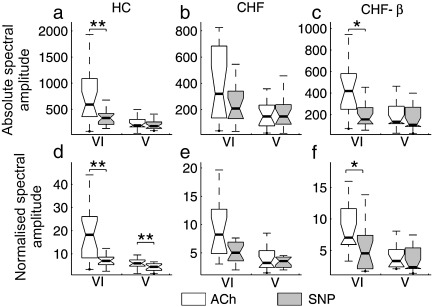
Effect of β1-blockers. The absolute spectral amplitude (upper row) and normalised spectral amplitude (lower row) are plotted for: (a, d) the HC group; (b, e) the CHF group prior to treatment; (c, f) the CHF group after treatment with β_1_-blockers. 0.01 < *p* < 0.05 is indicated by ⁎ and *p* < 0.01 by ⁎⁎. For an explanation of data presentation and error bars, see text (subsection on statistical analysis and presentation).

**Fig. 7:**
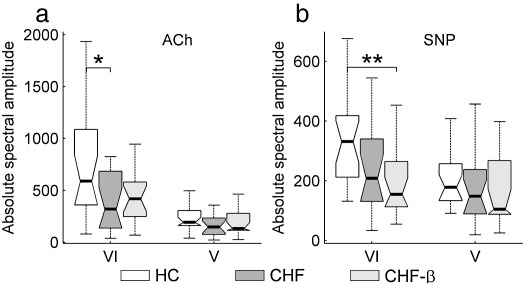
Comparison of the three subject groups (HC, CHF and CHF-β) in terms of their ACh and SNP responses. Absolute spectral amplitudes are presented in intervals V and VI for (a) ACh response and (b) SNP response. 0.01 < *p* < 0.05 is indicated by ⁎ and *p* < 0.01 by ⁎⁎. For an explanation of data presentation and error bars, see text (subsection on statistical analysis and presentation).

A nonpaired, nonparametric, Wilcoxon rank sum test was used to obtain the probability that two group distributions were equal (data presented in Figs. 4 and 5). A paired, Wilcoxon signed rank test was used to obtain the probability that the effect of two substances, ACh and SNP, was equal for a given group (data presented in Fig. 6). For hemodynamic data (Fig. 3) and skin temperature parametric *t*-tests were used, a nonpaired one in comparing patients with controls and a paired one between patient groups. Paired and nonpaired Wilcoxon tests were used to produce the statistics in Fig. 7. A nonpaired test was used to compare HC with patients before and after treatment, while a paired test was used to compare both patient groups.

In all hypothesis tests a value of *p *< 0.05 was considered significant. The graphical data in Fig. 3 and those that follow below are presented in each case as a box with lines drawn at the 25th percentile, the median and the 75th percentile. Whiskers extend from the ends of the box to the most extreme data value within 1.5 × IQR (where IQR is the inter-quartile range of the box). The range 0.01 < *p* < 0.05 is indicated by ⁎ and *p* < 0.01 by ⁎⁎. The statistical analysis was performed using Matlab Statistics Toolbox (The MathWorks, Inc.).

The demographic data in Table 1 are displayed as mean ± SD.

## Results

### Hemodynamic data

Treatment with β_1_-blockers resulted in a significant reduction in both systolic and diastolic blood pressure. The systolic pressure decreased from 141 ± 16 to 124 ± 18 mm Hg (*p* = 0.003). The diastolic pressure decreased from 77 ± 12 to 62 ± 10 mm Hg (*p* = 0.002).

The heart rate decreased significantly from 1.31 ± 0.27 to 0.95 ± 0.15 Hz (*p* = 0.0003). Although HR variability is not relevant to the main findings of this study, we noted that it decreased very slightly, mainly on account of less ectopy. For the group, the number of ectopic beats dropped from 4.1 to 1.2 per minute. With ectopics included, HR variability was significantly greater in the CHF group than in the HCs; when the analysis was repeated with the extra-systoles removed (as shown), there was no significant difference between HR variability in the HC and CHF groups.

For the first time we have demonstrated that neither the RF nor RF variability were significantly changed by treatment. Fig. 3 summarizes the heart and respiration statistics for all three subject groups, including the healthy controls. Note that, in the CHF group, HR, RF and RF variability are all significantly higher than for the age-matched HCs. Treatment had no statistically significant effect on either RF or RF variability.

### Skin temperature

There was a small decrease in patients' skin temperature as a result of the β_1_-blocker treatment (on arms 35.09 ± 1.49 °C before, 34.19 ± 1.18 °C after). The change is smaller than the variation between individual patients and is statistically insignificant.

### CHF compared to HC

Effect of ACh and SNP on mean value of the LDF signal and average spectral amplitude: a comparison of the HC and CHF groups' responses to ACh and SNP is presented in Fig. 4: (a) shows mean blood flow and (b) the average spectral amplitude. There are highly significant differences in the mean flows between these two subject groups, in their responses to both ACh and SNP. However, there are no statistically significant differences between them in the average spectral amplitudes of their responses to ACh and SNP.

Effect of ACh and SNP on oscillatory flow components: Fig. 5 presents data comparable to those of Fig. 4, but with added frequency discrimination, dividing the responses into the frequency intervals I–VI defined above. It is evident that almost all the oscillatory components are affected by CHF, but the effect on the lowest two seems to be different for the two vasodilators.

In more detail, there is a distinction between the CHF and HC groups in their absolute spectral amplitudes in interval VI, which is significant in response to ACh (Fig. 5a) but not to SNP (Fig. 5c). The same is true of their normalised spectral amplitudes (5b and d). Within intervals III and IV, there are highly significant differences between the CHF and HC groups (5a–d), but no distinction between their responses to ACh (5a,b) and SNP (5c,d). There is a significant difference between the normalized amplitudes for the two groups in interval V for ACh (5b).

### Effect of β_1_-blockers

The effect of Bisoprolol was evaluated in two different ways. First, the responses to ACh before and after the treatment were compared. Similarly, the comparison of responses to SNP before and after the treatment was performed. After treatment the response to ACh was slightly increased for each oscillatory component, but the increase was not statistically significant. The response to SNP did not change in any of the intervals.

Secondly, the responses to ACh and SNP were compared. The comparison was performed before the treatment and after the treatment as well as for the HC group. A significant difference between the responses to the two substances was found only within the lowest two frequency intervals and this is shown in Fig. 6.

The figure compares absolute and normalized spectral amplitudes in response to ACh and SNP for all three subject groups. Parts (a) and (d) illustrate the differences in response to ACh and SNP in the HC group. The difference in normalized amplitude in interval V has been demonstrated in several earlier studies (Kvernmo et al., 1999; Stefanovska et al., 1999; Kvandal et al., 2003; Stefanovska and Bračič, 1999, Kvandal et al., 2006). Note that there is, as recently discussed (Kvandal et al., 2006), a statistically significant difference also in interval VI.

Parts (b) and (e) of Fig. 6 illustrate the differences in response to ACh and SNP in the CHF group. There is no statistically significant difference in absolute (6b) and normalised spectral amplitude (6e) in the case of the CHF patients. Following treatment with β_1_-blockers, however, the CHF-β group exhibits differences in response to ACh and SNP in interval VI that are statistically significant, both in the absolute (6c) and in the normalised spectral amplitude (6f). The differences in response to ACh and SNP in interval V seen in the HC group are not, however, restored by the β_1_-blocker treatment.

Fig. 7 compares the three subject groups, HC, CHF and CHF-β directly in terms of their responses to ACh and SNP in intervals V and VI. In response to ACh in interval VI, there is a significant difference between HC and CHF but not between HC and CHF-β (Fig. 7a). In response to SNP there is a significant difference between HC and CHF-β in interval VI (Fig. 7b). No statistically significant difference between any of the three groups was observed in interval V, neither with ACh (Fig. 7a) nor with SNP (Fig. 7b).

## Discussion

The major findings in this study are that patients with CHF have significant abnormalities in endothelial function compared with healthy controls, and that treatment with Bisoprolol, a selective β_1_ antagonist, partially reverses the endothelial dysfunction that is found in patients with CHF. Whilst the mechanisms for the improvement in endothelial function are not explored in the present study the differences between intervals V and VI help to shed some light on the complex effects seen at the level of endothelial regulation. The changes in CHF as compared to healthy controls, and the effect of β_1_-blockers on endothelial function in CHF patients, are now discussed.

### Endothelial dependent oscillatory components

The fact that the oscillatory amplitudes in intervals V and VI are differentially affected by ACh and SNP demonstrates that activity in both intervals is associated with the endothelium. This finding was first reported in the work by Kvandal et al., 2006, and has been confirmed in the present study. Moreover, we have demonstrated that both intervals are abnormal in CHF as compared with healthy controls. Indeed, impaired endothelium-dependent vasodilatation and increased plasma concentration of a variety of neurohormones e.g. endothelin, angiotensin II, and natriuretic peptides in CHF, have been previously described (Vanhoutte, 1996). In addition, increased production of NO has been reported as an effect of β-blockers (Ignarro, 2004).

The reason for using iontophoresis for our administration of ACh and SNP was twofold. First, a comparison of responses to endothelium-dependent and endothelium-independent vasodilation provides a measure of endothelial activity. Secondly, vasodilator substances will, by mechanism of their action increase the mean value of the flow as well as all the oscillatory components. This is especially important in facilitating measurements of the low frequency oscillations which make a relatively low contribution to the flow in terms of energy.

Detailed information about the oscillations in these two intervals and their origin can be found in the Appendix A.

### CHF patients compared to healthy controls

The results in Fig. 5 clearly demonstrate that the responsiveness of the vascular endothelium in CHF patients is reduced compared to healthy controls. This finding has been well described in previous studies (Drexler et al., 1992; Morgan et al., 2004) of CHF patients. However, in those studies, invasive measurement techniques were used and/or the results were based on mean values of vascular responses, without the frequency discrimination. The current study, based on studying oscillations in addition to mean flows, has derived similar but more extensive information using entirely noninvasive techniques. Abnormalities of endothelial function in CHF are almost certainly multifactorial. Previous work has implicated NO through either decreased production (Bernstein et al., 1997), increased degradation of NO or decreased NO bioavailability (Belch et al., 1991). The expression of endothelial NO synthase (eNOS) has been shown to be reduced in both rats (Comini et al., 1996) and dogs (Smith et al., 1996) with experimental heart failure.

### CHF post β_1_-blockade

The differing responses of the CHF and CHF-β groups (Fig. 6) found in the present study show clear evidence that treatment with Bisoprolol brings an increase in endothelial responsiveness in frequency interval VI: the ACh/SNP differentiation in absolute and normalized spectral amplitude for untreated CHF patients is statistically insignificant (6b and 6e), whereas for the CHF-β group it is statistically significant (6c and 6f). Treatment with Bisoprolol evidently ameliorates the impairment of endothelial responsiveness associated with CHF.

In detail, the lack of ACh/SNP differentiation for CHF in interval VI can be explained by the fact that the response to ACh is significantly decreased in CHF compared to HC (Fig. 7a) while the SNP response is not (Fig. 7b). After treatment the ACh response increases compared to CHF (Fig. 7a), although not significantly. On the other hand the response to SNP is slightly reduced (Fig. 7b).

The mechanism of action of β_1_-blockers in CHF is still not wholly understood. Likely mechanisms involve reduction in sympathetic tone, increase in vagal tone, reduction of subendocardial ischaemia, reduction in rennin and endothelin release, increasing norepinephrine re-uptake, and reducing inflammatory cytokines (Silke, 2006). It has been reported in several studies that ventricular function, measured by left ventricular ejection fraction (LVEF), improves as a result of β-blocker treatment in CHF (Van Campen et al., 1998). Whether the increased LVEF might have an indirect effect on our findings is not known and further work will have to be done.

We note that β_1_-blockers operate in many different ways, and it has yet to be determined which of them is/are responsible for the observed changes in low frequency blood flow spectra.

What the present study has shown is that CHF patients exhibit blood flow abnormalities in frequency interval VI, that iontophoresis and wavelet analysis enable this abnormality to be detected, and that the medication partially restores normal function.

### Study limitations

As with all clinical studies involving patient follow up over time, it was not feasible to accommodate for the effects of disease progression. In addition all patients were on medication at the commencement of the study, and alteration of doses of other drugs e.g. diuretics was sometimes necessary at the discretion of the prescribing physician.

The study was not performed in a placebo-controlled fashion because it would have been ethically unacceptable to withhold a life-prolonging treatment from 50% of the patients for 12 weeks.

The main reason for the introduction of normalised spectral amplitudes is to reduce the inter-subject variability seen in the case of absolute amplitudes. However, an effect on normalised amplitude within a specific interval can then result from changes in the rest of the spectrum, not only from changes in the interval under consideration. For this reason special care should be taken when interpreting normalised data. In the present study, the main findings are based on comparison of responses to ACh and SNP within frequency intervals V and VI (Fig. 6). Average spectral amplitudes for both substances did not differ between any of the subject groups and differences between the effects of the substances were found only in the two lowest frequency intervals. This suggests that oscillations at higher frequencies, such as those related to heartbeat and respiration did not alter the normalised spectral amplitudes at low frequencies.

### Conclusions

The main conclusion is that, compared to healthy controls, CHF patients exhibit abnormally attenuated blood flow oscillations in frequency intervals V and VI, which represent different aspects of endothelial function. Treatment with Bisoprolol shifts the abnormality in spectral amplitude back towards the findings in healthy controls.

In more detail, iontophoretic administration of the endothelium-dependent and endothelium-independent vasodilators ACh and SNP has confirmed that the low-frequency spectral peaks in intervals V and VI of the LDF blood flow signal are associated with endothelial reactivity. Both peaks are significantly attenuated in patients with CHF. These spectral peaks are respectively governed by NO, and an as yet unknown factor. Treatment of CHF patients with the β_1_-blocker Bisoprolol moves the spectral amplitude of interval VI closer to that of the healthy controls. However, given that no significant change of activity in interval V is detected as a result of the treatment, whereas a highly significant change is observed in interval VI, it can be inferred that the effects of Bisoprolol on the endothelium are at least partly mediated through a non-NO mechanism.

## Figures and Tables

**Table 1:** Clinical characteristics of the CHF study population before treatment

Age, y	69 ± 10
Sex	11 male, 6 female
Aetiology of CHF, *n*	
Ischaemia	6
Hypertension	2
Valvular heart disease	3
Idiopathic dilated cardiomyopathy	6
Heart rate, bpm	79 ± 16
Blood pressure, mm Hg	
Systolic	141 ± 16
Diastolic	77 ± 12
Total cholesterol, mmol/l	5.0 ± 1.2
Length of treatment, weeks	20 ± 10
Time to maximum tolerated medication, weeks	15 ± 6

Group mean and standard deviation are provided where relevant.

**Table 2:** Frequency intervals

Interval	Frequency (Hz)	Physiological origin
I	0.6–2.0	heartbeat
II	0.145–0.6	respiratory activity
III	0.052–0.145	intrinsic myogenic activity
IV	0.021–0.052	neurogenic (sympathetic) activity
V	0.0095–0.021	NO-dependent endothelial activity
VI	0.005–0.0095	non-NO-dependent endothelial activity

## Appendix A Oscillations in the LDF time series and their physiological origin

The dynamics of the microcirculatory flow, measured by LDF, consists of rhythmic variations. It can therefore be analysed using spectral techniques. The strengths of individual oscillating components, revealed by the spectrum, provide information about the dynamics of vascular regulatory mechanisms.

In earlier work, wavelet analysis of 20-minute LDF recordings using the Morlet mother wavelet resolved five oscillations in the time-frequency domain, the lowest one around 0.01 Hz (Bračič and Stefanovska, 1998). By tracing the characteristic frequencies of oscillations in time, the spectrum was divided into five intervals, such that only one oscillation was present in each interval (Bračič and Stefanovska, 1998; Stefanovska et al., 1999; Stefanovska and Bračič, 1999). Longer recordings enabled investigations of even lower frequency components in LDF signals and from 30-minute recordings another spectral component was observed at frequency around 0.007 Hz (Kvandal et al., 2006). It was shown not to be an artefact related to the measurement or to signal processing technique.

The positions of the characteristic frequencies vary slightly, with time and between subjects. They are also changed during physical activity, and are modified in some diseases. An ideal division of the spectrum into frequency intervals is therefore impossible. However, specific physiological processes appear to dominate in specific frequency intervals, and wavelet-based analysis provides a promising way of identifying them, especially for low frequency oscillations. Several recent studies have independently confirmed the presence of the oscillations in LDF recordings (Geyer et al., 2004; Humeau et al., 2004; Liu et al., 2006; Rossi et al., 2006; Li et al., 2006; Stewart et al., 2007; Hafner et al., 2007) and investigated their physiological origin. However, further work is necessary to fully understand the complex mechanisms involved in the regulation of the microvascular blood flow.

These mechanisms were recently reviewed by Segal (2005) who writes “With vasomotor tone reflecting myogenic contraction of smooth muscle cells modulated by shear stress on the endothelium, the initiation of functional vasodilation and its modulation by sympathetic innervation dictate how and where blood flow is distributed in response to metabolic demand”. Although metabolic demand can be strongly enhanced by exercise, it is of course continuously present in a living system. Moreover, the oscillatory nature of the flow generated by rhythmic activity of the heart can be expected to be associated with oscillatory mechanisms of regulation. Each of the regulatory mechanisms manifests within a particular frequency interval. In what follows we list the six previously defined (Kvandal et al., 2006) frequency intervals and we summarise the rationale of associating them with specific physiological processes.

### Frequency interval I (0.6–2 Hz), related to the heartbeat and frequency interval II (0.145–0.6 Hz), related to the respiratory activity

The oscillation with the highest frequency, around 1 Hz, is related to the activity of the heart. The respiratory activity is around 0.25 Hz. Both oscillatory components appear in the spectrum of the LDF signal and their physiological origin can easily be demonstrated by comparing the frequency content of ECG, respiration and blood pressure signals with that of a simultaneously measured LDF blood flow signal.

### Frequency interval III (0.052–0.145 Hz), related to intrinsic myogenic activity

In 1964 Folkow suggested that oscillatory changes of arteriolar diameter are resulted from myogenic control (Folkow, 1964). Several years later, Johnson (1991) showed that blood vessels respond to transmural pressure elevation with constriction, and to pressure reduction with dilation. He termed this behaviour myogenic response and suggested that it is inherent to smooth muscle and independent of neural, metabolic, and hormonal influences. It was shown that this response is not constant for all the vessels and that a longitudinal gradient in myogenic responsiveness exists within an arteriolar network (Davis, 1993).

Spontaneous activity recorded in microvascular smooth muscle cells was shown to lie in the range 4–10 events a minute (0.07–0.1 Hz) (Golenhofen, 1970). Several studies suggested that these waves are of local origin representing intrinsic myogenic activity of smooth muscle cells in resistance vessels (Salerud et al., 1983; Colantuoni et al., 1984; Meyer et al., 1988; Intaglietta, 1989; Kastrup et al., 1989; Hoffmann et al., 1990; Bollinger et al., 1991). Rhythmical variation in the same frequency interval (6 to 10 cycles a minute) had been observed in blood pressure recordings by Traube (1865) and was confirmed by Hering (1869). In 1876 Siegmund Mayer observed similar oscillations (Mayer, 1876). Killip (1962) presented evidence that Mayer waves are accompanied by rhythmic fluctuations in vascular resistance.

In a recent study on small arteries in rats, it was shown that blockade of adrenoceptors, which mediate the effect of the main transmitter in these vessels, does not alter the myogenic response (Anschutz and Schubert, 2005). Similar findings have been reported after inhibition of nerve action potentials by tetrodotoxin, after blockade of postsynaptic receptor function by receptor antagonists and after chemical vessel denervation in cat cerebral (Harder, 1984), rat cerebral (Osol and Halpern, 1985), and rat saphenous (Berczi et al., 1992) arteries. These studies provide supporting evidence that the myogenic response is due to an action of transmural pressure on smooth muscle cells only. This is in agreement with the study in humans, where blood flow oscillations from free microvascular flaps deprived of sympathetic nerve activity (SNA) and from intact skin were compared (Söderström et al., 2003). It was shown that the SNA was not manifested in the frequency interval above 0.05 Hz.

Wavelet analysis of LDF signals was used to trace the myogenic activity related oscillation in time and attribute it to a specific frequency interval. The technique was not used to investigate the physiological origin of this oscillation, which had already been revealed from both blood flow and blood pressure signals, as described above.

The application of wavelet analysis to blood flow signals has revealed that the amplitude of myogenic oscillations is increased by exercise (Kvernmo et al., 1998; Hubscher et al., 2006) and decreased by local cooling (Bandrivskyy et al., 2004b).

### Frequency interval IV (0.021–0.052 Hz), related to neurogenic (sympathetic) activity

An oscillation with a characteristic frequency around 0.04 Hz has been observed in blood pressure, blood flow and HRV signals. It was attributed either to metabolic (Kitney et al., 1985) or to neurogenic activity (Stefanovska et al., 1997), which is superimposed on myogenic activity in the regulation of the blood pressure through adjustment of the vessel's radius.

Kastrup et al. (1989) termed these rhythmic variations β-oscillations and, in agreement with the study of Golenhofen and Hildebrandt (1958), showed that in humans they disappear after local and ganglionic nerve blockade and in chronically sympathectomized tissue. They suggested that β-oscillations are a vascular reaction of purely neurogenic origin. Similarly, in an LDF study of oscillations in rabbit skeletal muscle tissue, slow waves with a frequency of around 1–3 per minute were suggested to be under neurogenic control since they disappeared after pharmacological nerve blockade (Schmidt et al., 1992).

Using wavelet analysis with a logarithmic frequency resolution (Stefanovska et al., 1999) several studies confirmed the involvement of sympathetic nerve activity in this low frequency interval. Analysis of skin blood flows on rats' paws has shown a significant decrease in the relative energy contribution to blood flow oscillations in the low frequency spectrum only in denervated paws (Bajrović et al., 2000). In humans, LDF signals from the surfaces of free microvascular flaps, deprived of sympathetic nerve activity, showed reduced spectral amplitude compared to adjacent intact skin only in the 0.02–0.05 Hz low frequency interval (Söderström et al., 2003). Two recently published studies have shown the 0.021–0.052 Hz frequency interval to be related to sympathetic activity: after brachial plexus block, a significant decrease was shown in the lower forearm skin compared to controls (Landsverk et al., 2006), a result that was confirmed on a similar location as an effect of general anaesthesia (Landsverk et al., 2007).

An independent study has confirmed these findings in the same frequency interval by simultaneous measurements of LDF signals on the surfaces of a free latissimus dorsi myocutaneous flap and on the adjacent intact skin of a healthy limb (Liu et al., 2006).

### Frequency interval V (0.0095–0.021 Hz), related to endothelial activity (partly NO-dependent)

It is well accepted that ACh induces vasodilatation through enhancement of the activity of endothelial cells, but the exact mechanism and the mediators involved are still not fully understood. The involvement of endothelium in ACh-induced vasodilatation is the main difference as compared to vasodilatation by SNP. Therefore, iontophoresis with these two substances has been used to examine skin microvascular endothelial cell function (Westerman et al., 1988; Enerothgrimfors et al., 1993; Morris and Shore, 1996). It was suggested (Moncada et al., 1991) that impaired ACh-induced vasodilatation, as compared to the vasodilatation induced by SNP, could be taken as a demonstration of endothelial dysfunction.

Following reports of endothelial and NO involvement in rhythmic activities, the hypothesis was tested that not only the mean value of the blood flow, but also its dynamics is modulated by endothelium (Kvernmo et al., 1999). Using wavelet analysis of 20-minute LDF recordings with logarithmic frequency resolution, the difference between ACh and SNP responses was found to manifest in only one frequency interval, around 0.01 Hz. The oscillation in this interval has been attributed to endothelium-mediated vasodilatation but its physiological origin has not been investigated. These findings were confirmed in a separate study on athletes and healthy control subjects using the same dose–response protocol (Kvernmo et al., 2003).

Observation of an oscillatory component in the spectrum at an even lower frequency, around 0.007 Hz, has led to 30-minute recordings of LDF signals and an iontophoresis protocol consisting of 7 equal pulses. The protocol was carefully chosen to avoid current induced vasodilation (iontophoretic currents less than 200 μA and total charge less than 8 mC) (Westerman et al., 1988). Possible effects of the iontophoresis current (Veber et al., 2004) and transdermal potential difference (Bandrivskyy et al., 2004a) on the blood flow responses were investigated and were shown not to be involved in the low frequency oscillations.

In further investigation of the low frequency oscillations, several other studies have confirmed the role of endothelium in frequency interval V (Kvandal et al., 2003, 2006; Landsverk et al., 2006, 2007) including the present study on CHF. It was suggested that oscillations in this interval were partly mediated by NO, as they were reduced by infusion of l-NMMA and restored by l-arginine, but were not mediated by endogenous prostaglandins (Kvandal et al., 2003). The finding that oscillations within this frequency interval are NO-dependent was confirmed in a recent independent study (Stewart et al., 2007).

Oscillations in interval V were significantly reduced following both brachial plexus block (Landsverk et al., 2006) and general anaesthesia (Landsverk et al., 2007). Although the difference in responses to ACh and SNP in this interval was abolished after plexus block (Landsverk et al., 2006), this was not the case following general anaesthesia (Landsverk et al., 2007). The possible reason for this finding is that smooth muscle cell activity is reduced under general, but not under local, anaesthesia.

### Frequency interval VI (0.005–0.0095 Hz), related to endothelial activity (non-NO-dependent)

As within frequency interval V, the proportion of the flow in frequency interval VI was shown to be significantly higher in response to ACh compared to SNP, indicating an endothelium related origin of the oscillation (Kvandal et al., 2006). However, blocking the endothelial production of NO and prostaglandins did not affect this oscillation, suggesting possible involvement of other endothelial mechanisms.

The possible involvement of endothelium in this frequency interval has also been demonstrated and discussed above in the present CHF study.
